# Non-invasive Autonomic Neuromodulation Is Opening New Landscapes for Cardiovascular Diseases

**DOI:** 10.3389/fphys.2020.550578

**Published:** 2020-12-15

**Authors:** Mingxian Chen, Songyun Wang, Xuping Li, Lilei Yu, Hui Yang, Qiming Liu, Jianjun Tang, Shenghua Zhou

**Affiliations:** ^1^Department of Cardiology, The Second Xiangya Hospital of Central South University, Changsha, China; ^2^Department of Cardiology, Renmin Hospital, Wuhan University, Wuhan, China

**Keywords:** heart, cardiovascular disease, autonomic nervous system, neuromadulation, non-invasive

## Abstract

Autonomic imbalance plays a crucial role in the genesis and maintenance of cardiac disorders. Approaches to maintain sympatho-vagal balance in heart diseases have gained great interest in recent years. Emerging therapies However, certain types of emerging therapies including direct electrical stimulation and nerve denervation require invasive implantation of a generator and a bipolar electrode subcutaneously or result in autonomic nervous system (ANS) damage, inevitably increasing the risk of complications. More recently, non-invasive neuromodulation approaches have received great interest in ANS modulation. Non-invasive approaches have opened new fields in the treatment of cardiovascular diseases. Herein, we will review the protective roles of non-invasive neuromodulation techniques in heart diseases, including transcutaneous auricular vagus nerve stimulation, electromagnetic field stimulation, ultrasound stimulation, autonomic modulation in optogenetics, and light-emitting diode and transcutaneous cervical vagus nerve stimulation (gammaCore).

## Introduction

Cardiovascular diseases are the leading cause of death with high morbidity and mortality (Roth et al., 2017). Autonomic nervous system (ANS) imbalance is associated with disease progression and negative clinical outcomes (Shen and Zipes, 2014; Lai et al., 2019). It usually participates in the genesis and maintenance of various cardiovascular diseases, including heart failure, arrhythmias, acute myocardial infarction, and hypertension. However, cardiac disorders can in turn further aggravate the imbalance of the ANS, resulting in a vicious cycle between autonomic imbalance and cardiovascular diseases (Chen et al., 2019). Therefore, the ANS has been regarded as an important target to break this vicious cycle (Chen et al., 2015a,b). Due to the limited effectiveness of pharmacologic agents on ANS regulation, device-based neuromodulation has led to interest in applications for cardiovascular disorders. Additionally, cardiac neuromodulation has been successfully performed to modulate the cardiac ANS in the treatment of these disorders by extensive research (Sohinki and Stavrakis, 2019; Waldron et al., 2019).

Approaches via electrical devices or nerve denervation are mainly designed to modulate ANS activity and have become an emerging therapeutic strategy for the treatment of cardiovascular diseases (Cook et al., 2014). According to anatomical placement or nerve denervation, neurostimulation therapies can be divided into invasive and non-invasive approaches. Invasive or minimally invasive therapeutic approaches include cervical vagal stimulation (Chen et al., 2016), baroreceptor activation therapy (Sheng et al., 2016), spinal cord stimulation (Lopshire and Zipes, 2014), ganglionated plexi ablation (Zipes, 2017), ganglionated plexi stimulation (Wang S. et al., 2015), renal sympathetic nerve denervation (Yu et al., 2017a,b), and left cardiac sympathetic nerve denervation (Cha et al., 2019). However, these approaches sometimes unavoidably increase the potential risk of hardware-related complications and serious adverse events, including permanent neurological damage. Importantly, electrodes surrounding the nerve trunk sometimes produce scar tissue (fibrosis), which can potentially increase the stimulation threshold. These complications may contribute to the translation failure of some types of device-related stimulation (Olshansky, 2016; Sahyouni et al., 2017).

Recently, non-invasive neuromodulation approaches have received great interest in ANS modulation for the treatment of cardiovascular diseases. The “non-invasive” techniques in this review refer to interventions that do not use electrodes around the nerve trunk or cause damage to nerve fibers. Importantly, the advantages of non-invasive approaches, such as low cost, portability, and ease of use, have promoted rapid evolution in recent decades (Schluter et al., 2018). Furthermore, non-invasive approaches are an attractive option for clinicians as novel therapies and are highly recommended for the treatment of cardiovascular diseases. Non-invasive neuromodulation has gradually and widely been applied in experimental evidence and clinical trials of heart diseases. In this review, we discuss the protective impact of non-invasive neuromodulation on the treatment of cardiovascular diseases.

## Cardiac Autonomic Nervous System and Invasive Neuromodulation

**Figure 1:**
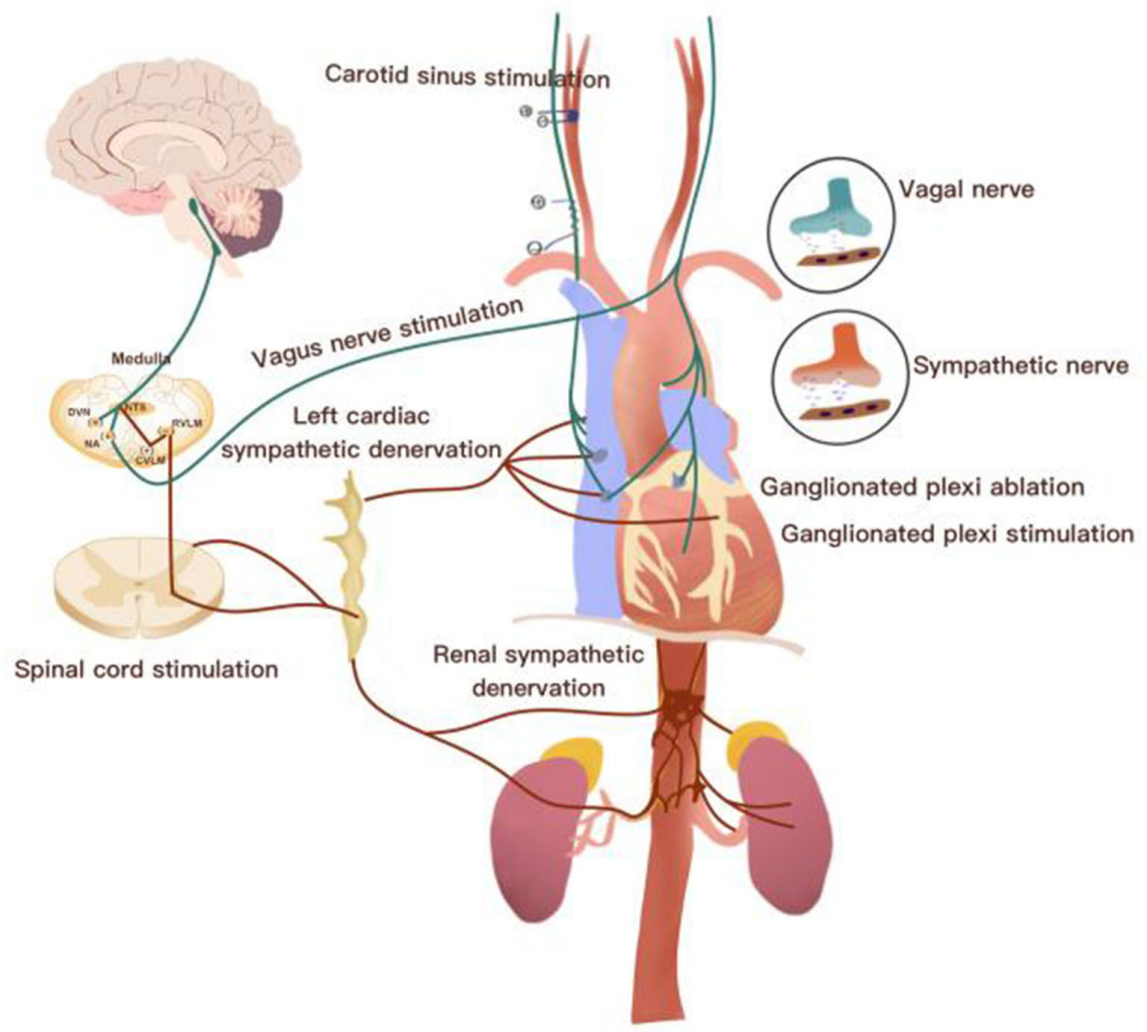
Overview of the neurocardiac axis and invasive neuromodulatory approaches. CVLM, caudal ventrolateral medulla; DVN, dorsal vagal nucleus; IML, intermediolateral cell column; NTS, nucleus of the solitary tract; RVLM, rostroventrolateral medulla.

The cardiac autonomic nervous system (CANS) consists of an extrinsic autonomic cardiac nervous system (EACNS) and an intrinsic autonomic cardiac nervous system (ICANS). The EACNS comprises fibers that mediate connections between the heart and the nervous system, whereas the ICANS consists of primarily autonomic nerve fibers once they enter the pericardial sac (Figure 1) (Zipes, 2008; Wickramasinghe and Patel, 2013; Chen et al., 2014).

The ECANS is made up of sympathetic and parasympathetic fibers. The sympathetic fibers are mainly derived from cervical ganglia, stellate ganglia, and thoracic ganglia along with the spinal cord; these fibers form the superior, middle, and inferior cardiac nerves and terminate on the surface of the heart (Witt et al., 2017). Renal sympathetic nerves (RSNs), which include efferent and afferent fibers, are adjacent to the wall of the renal artery and crucial for the production of catecholamines contributing to hypertension. Studies have shown an association between the left stellate ganglion (LSG) and RSNs. Afferent RSNs can affect the nerve activity of the LSG by modulating central sympathetic outflow. The nucleus ambiguous of the medulla oblongata delivers parasympathetic fibers predominantly into the vagal nerve (Ripplinger et al., 2016). The terminals of the vagal nerve relay to the fat pad of the heart and form the ganglion plexus (GP), mainly called the ICANS. GPs are divided into atrial and ventricular GPs located on the surface of the heart. GPs are the integration centers that modulate the intricate autonomic interactions between the ECANS and ICANS (Chen et al., 2014; Brack, 2015).

Afferent fibers are divided into vagal and sympathetic afferents (Burger and Verkuil, 2018). Cardiovascular receptors, including chemoreceptors and baroreceptors, transmit signals from cardiac activity or tissue injury to afferent cardiac nerves. Cardiac afferent fibers deliver signals to the nucleus tractus solitaries (NTS) and dorsomedial spinal trigeminal tract (Zoccal et al., 2014). After NTS receiving information, it projects to caudal ventrolateral medulla (CVM) and rostral ventrolateral medulla (RVM) to coordinate the activity of sympathetic nervous system, and then it finally leads to decrease sympathetic tone outflow. At the same time, NTS also projects information to the dorsal motor nucleus (DMN) of the vagus and contributes to vagal nerve activity enhancement (Ricardo and Koh, 1978). Currently, emerging therapies, including vagus nerve stimulation, baroreflex activation therapy, spinal cord stimulation, renal nerve denervation, left stellate ganglion ablation, GP ablation, and GP stimulation, have been widely applied in cardiovascular diseases (Clancy et al., 2014; Zubcevic et al., 2019).

## The Potential Risks of Invasive Neuromodulation

Device-related neuromodulation consists of a generator and electrodes. Implantation of the generator and electrodes requires surgery, especially for children; this approach requires a general anesthetic and at least an overnight stay in the hospital. For instance, the electrodes of some device-based stimulation are designed to surround the nerve trunk to deliver electrical information. A fibrotic scar around the interface induced by the inflammatory response can increase impedance. This leads to the inefficient transduction of electrical signals (Eldabe et al., 2016; Lotti et al., 2017).

The application of invasive neuromodulation is also limited by a variety of potential complications. Complications can be divided into device-related and biologic aspects (Doruk Camsari et al., 2018). Device-related complications include lead fracture or migration, intermittent stimulation, over- or under-stimulation, loose connections, hardware malfunction, battery replacement, and communication failure with the generator (Levy, 2013). Biologic complications consist of epidural hemorrhage, infection, voice disturbances, cough, headache, paralysis, cerebrospinal fluid leakage, pain over the implant site, allergic reactions, skin breakdown, surgical costs, and the need for post-operative monitoring (Levy et al., 2011; Shamji et al., 2015; Petraglia et al., 2016).

Non-invasive neuromodulation is a relatively new and promising method with potential advantages as an alternative to invasive neuromodulation. Non-invasive neuromodulation is easier and less invasive than traditional neuromodulation, thus reducing the risk of complications (Eldabe et al., 2016; Cotero et al., 2019).

## Non-Invasive Neuromodulation and Cardiovascular Diseases

**Figure 2:**
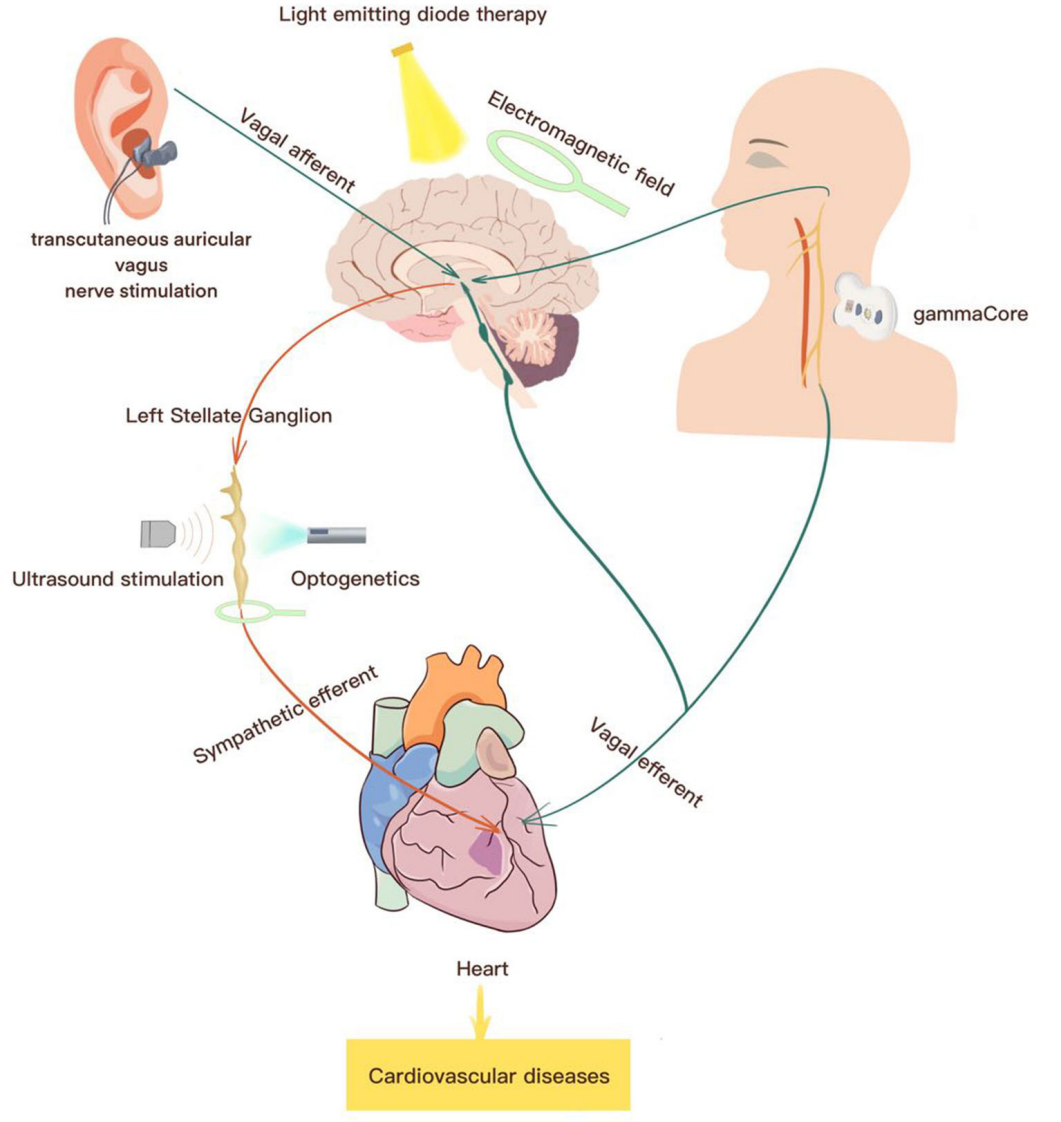
Summary of potential non-invasive neuromodulations for the treatment of cardiovascular diseases. Current therapeutic strategies for the treatment of cardiovascular diseases include transcutaneous auricular vagus nerve stimulation, electromagnetic field stimulation, ultrasound stimulation, optogenetics for stimulation, transcutaneous cervical nerve stimulation.

Electrical device stimulation and sympathetic nerve denervation have progressively been a focus of non-pharmaceutical approaches for the treatment of cardiovascular diseases (Lohmeier and Hall, 2019). Recently, current non-invasive neuromodulation techniques have gained particular interest in cardiovascular disorders. They not only provide deep insight into autonomic circuit physiology but also can be applied for therapeutic purposes (Table 1 and Figure 2).

**Table 1:** Studies of non-invasive autonomic neuromodulations in the treatment of cardiovascular diseases.

**Modulation**	**References**	**Species**	**Parts**	**Parameters**	**Models**	**Results**
Tragus nerve stimulation	Yu et al. (2013)	Canine	Ear	The voltage of 80% below the threshold	Atrial fibrillation	Inhibit AF and electrophysiological changes
	Chen et al. (2015c)	Canine	Right Ear	The voltage of 80% below the threshold	Atrial fibrillation	Inhibit AF by the expression of connexs
	Chen et al. (2015d)	Canine	Left-sided tragus	The voltage of 80% below the threshold	Atrial fibrillation	Inhibit AF by conexin expression
	Wang et al. (2014)	Canine	Left-sided tragus	The voltage of 80% below the threshold	Chronic myocardial infarction	Ameliorate ventricular remodeling and function
	Wang S. et al. (2015)	Canine	Left-sided tragus	The voltage of 80% below the threshold	Chronic myocardial infarction	Improve ventricular remodeling by the expression of collagen, TGF-β, MMP-9
	Yu et al. (2016)	Canines	Left-sided ear	The voltage of 80% below the threshold	Chronic myocardial infarction	Inhibit LSG activity and remodeling
	Zhou et al. (2016)	Canine	Right tragus	The voltage of 80% below the threshold	Ligh-frequency stimulation of RSG	Suppress RSG activity and sympathetically induced sinus node acceleration
	Zhou et al. (2019)	Rats	Tragus	(20 Hz, 2 mA, 0.2 ms) was implemented for 30 min daily for 4 weeks	Heart failure with preserved ejection fraction	Ameliorate diastolic dysfunction, and attenuate cardiac inflammation and fibrosis
	Nasi-Er et al. (2019)	Dogs	Bilateral Ears	50% of the threshold voltage	Acute myocardial infarction	Attenuate electrophysiological changes and ventricular structural remodeling, inhibit VAs
	Stavrakis et al. (2015)	Patients	Ear			
	Yu et al. (2017c)	Patients	Ear	50% lower than the electric current that slowed the sinus rate	Ischemia reperfusion injury	Reduce VAs Attenuate cardiac function
Magnetic field stimulation	Wang et al. (2016)	Canine	Left stellate ganglion	1 HZ; stimulation time 8 s; interstimulus interval, 5 s	Acute myocardial infarction	Inhibit LSG activity and VAs
	Nishikawa et al. (2017)	Patients	Right cervical vagus nerve	Frequency 5 or 20 Hz, pulse to 250 us	Healthy	No side effects without bradycardia and arterial pressure changes
Ultrasonic stimulation	Wang et al. (2019a)	Dogs	LSG	Frequency 1 MHz, Pulse repetition frequency 1 KHz, Duty cycle 8 s, Duty cycle % 50%	Acute myocardial infarction	Suppress LSG activity and VAs
Optogenetic stimulation	Yu et al. (2017d)	Canine	LSG	Light-emitting diode illumination (565 nm)	Myocardial infarction	Inhibit LSG activety and VAs
Light-emitting diode	Wang et al. (2019b)	Rats	Brain	Peak wavelength, 610 nm; power intensity, 1.7 mW/cm^2^; energy density, 2.0 J/cm^2^	Acute myocardial infarction	Inhibit LSG activity and VAs. Reduce inflammation
	Wang et al. (2019c)	Rats	Brain	Peak wavelength, 610 nm; power intensity, 1.7 mW/cm^2^; energy density, 2.0 J/cm^2^	Acute myocardial ischemia reperfusion injury	Inhibit LSG activity and VAs. Reduce inflammation

*LSG, left stellate ganglion; MMP, matrix metalloproteinase; RSG, right stellate ganglion; TGF, transforming growth factor; VAs, ventricular arrhythmias*.

### Transcutaneous Auricular Vagus Nerve Stimulation and Cardiovascular Diseases

The auricular branch of the vagus nerve (ABVN), a powerful nerve entering the brain, is the only afferent peripheral branch of the vagus nerve that is located in the skin. The ABVN delivers information to the central ANS. After integration, it finally causes an increase in vagal nerve activity and a reduction in sympathetic tone. Transcutaneous auricular vagus nerve stimulation (ta-VNS) is a non-invasive method applied to electrically regulate vagal tone and brain activity by the vagal afferent pathway (Kaniusas et al., 2019a,b). Clancy et al. (2014) demonstrated that ta-VNS could increase parasympathetic predominance assessed by heart rate viability and could reduce sympathetic nerve activity detected by microneurography. Decades ago, ta-VNS was first applied for cognitive, emotional, and neurological modulation, with a similar effect as invasive cervical vagus nerve stimulation. In recent years, because of the novel and non-invasive technology, ta-VNS has been applied in both research and therapy as a medical treatment tool for cardiovascular diseases (Shiozawa et al., 2014; Redgrave et al., 2018).

Recently, as a type of non-invasive neuromodulation, ta-VNS has been gradually and widely applied to treat cardiac diseases, including atrial fibrillation (AF), acute and chronic ischemia diseases, and heart failure induced by autonomic imbalance. Previous studies have shown that high-intensity vagus nerve stimulation induces HF, but low-level vagus nerve stimulation (LL-VNS) has been proven to suppress AF (Zhang et al., 2009; Sheng et al., 2011; Shen et al., 2013). Low-level ta-VNS (LL-ta-VNS) has the same effect as LL-VNS. Yu et al. (2013) first reported that LL-ta-VNS could reverse RAP-induced atrial remodeling and inhibit AF inducibility in canine models. This indicates that LL-ta-VNS is a potential non-invasive approach for AF treatment. We further found that LL-ta-VNS inhibited AF by modulating the expression of atrial connexin40 and connexin43 (Chen et al., 2015c,d). Later, Stavrakis et al. (2015, 2017) showed that LL-ta-VNS suppressed AF and decreased inflammatory cytokines in patients with paroxysmal AF in a clinical study. Later, they continued to conduct a TREAT AF trial to prove the long-term effect of LL-ta-VNS.

Beyond its atrial protective effects, LL-ta-VNS has also been applied to research left ventricular remodeling and arrhythmias. Wang et al. (2014) and Wang Z. et al. (2015) showed that chronic intermittent LL-ta-VNS could attenuate left ventricular remodeling in conscious dogs with healed myocardial infarction. It demonstrated ventricular protection against ta-VNS. Chronic LL-ta-VNS could reduce the inducibility of ventricular arrhythmia, LSG neural activity, and sympathetic neural remodeling in a post-infarction canine model. Downregulation of nerve growth factor protein and upregulation of SK2 protein in the LSG contributed to the salutary effects of LL-ta-VNS (Yu et al., 2016). Nasi-Er et al. also demonstrated that TS reduced the occurrence of spontaneous ventricular arrhythmias in conscious dogs with MI. The potential mechanisms of anti-ventricular arrhythmias may increase ventricular electrical stability and alleviate ventricular interstitial fibrosis (Nasi-Er et al., 2019). In a recent patient study, Yu et al. (2017c) found that LL-ta-VNS reduced the incidence of reperfusion-related ventricular arrhythmias during the first 24 h after acute myocardial infarction and improved left ventricular function 5–7 days after reperfusion along with reduced venous cytokine levels.

Sympathetic activity can regulate sinus node acceleration. Zhou et al. (2016) performed a study and showed that right-sided ta-VNS could inhibit sinus node acceleration induced by sympathetic nerves. Its potential mechanism may suppress right-sided stellate ganglion activity by modulating SK2, c-fos, and nerve growth factor protein expression. Currently, there is limited evidence to prove that drugs can improve the outcomes of heart failure with preserved ejection fraction. Interestingly, Po et al. also demonstrated that LL-ta-VNS could effectively treat heart failure with preserved ejection fraction in a rat model. It attenuated cardiac remodeling by inhibiting left ventricular fibrosis and inflammatory cell infiltration (Zhou et al., 2019). However, further trials are needed to study and support the observed beneficial effects in clinical settings.

However, because of the absence of standards regarding stimulation protocols, the parameters of ta-VNS have not been used consistently in research (Borges et al., 2019). Several studies have not shown positive effects of ta-VNS on vagal-related heart rate viability. The heterogeneous results may be due to the use of different stimulation parameters, differing in electrode placement areas on the ear, pulse width, frequency, and on–off cycle (Burger et al., 2016, 2017, 2019; De Couck et al., 2017).

### Electromagnetic Fields in the CANS and Cardiovascular Diseases

Electromagnetic waves are waves caused by vibrations between an electric field and a magnetic field. Electromagnetic waves transfer energy to tissues, resulting in functional changes or structural damage. Electromagnetic fields (EMFs) were first explored for use in diagnosing human diseases. In recent years, because of their non-invasive and safe advantages, EMFs have been developed and applied to treat a wide range of diseases, such as nervous system disorders, cardiovascular diseases, diabetes, spinal cord injuries, ulcers, and asthma (Schestatsky et al., 2013; Cabrerizo et al., 2014; Vernieri et al., 2014; Chervyakov et al., 2015).

Interestingly, EMF exposure can affect the structure and modulate the function of the ANS. EMFs can significantly change the physiological properties of the CANS neural network by the results of some ionic flux changes. A study showed that EMF exposure could increase sympathetic vasoconstrictor activity (Braune et al., 2002). Different parameters (frequencies and amplitudes) of stimulation contribute to different results. Recently, Wang et al. (2016) showed that EMF stimulation located on the surface of the left stellate ganglion could effectively reduce sympathetic activity and the incidence of ventricular arrhythmias in myocardial infarction canine models. EMF stimulation was applied with low frequency (1 Hz, intensity at ~90% of the motor threshold; 8 s on, 10 s off). EMF stimulation of the left stellate ganglion is a novel therapeutic strategy for treating ventricular arrhythmias associated with autonomic imbalance. Among EMFs for the vagus nerve, Nishikawa et al. (2017) found that EMFs could induce transient heart rate reduction in some healthy individuals but failed to induce sustained bradycardia and arterial pressure changes. This indicates that the magnetic focus and optimized stimulation need further improvement for beneficial effects in acute myocardial infarction. Scherlag et al. used a low-frequency electromagnetic field (LL-EMF) to expose the chest for 35 min in canine AF models and found that LL-EMF could successfully inhibit AF for 3–4 h (Yu et al., 2015). As is well-known, pericardial fat pads, including ganglion plexi, exist in high numbers on the surface of the heart. Whether LL-EMFs suppress AF by affecting the ganglion plexus remains unknown. There is still a need for further studies to prove this influence.

EMFs have the advantages of simplicity, low operating costs, and unproven harmful effects. EMFs show promising potential in the treatment of cardiovascular diseases by autonomic neuromodulation.

### Ultrasound Stimulation of the CANS and Cardiovascular Diseases

In the 1950s, ultrasound was applied to visualize tissue structure for diagnostic applications (Edler and Lindstrom, 2004). Ultrasound was also used for therapeutic indications as an ablative approach for the treatment of Parkinson's disease in earlier decades (Leinenga et al., 2016). Therapeutic ultrasound stimulation technologies are currently approved by the United States Food and Drug Administration (FDA) and used for the treatment of multiple diseases. Ultrasound stimulation parameters (frequency, amplitude, pulse duration) can be optimized for therapeutic or diagnostic applications (Downs et al., 2018). Ultrasound waves are transmitted into tissues. A portion of the waves are converted into thermal energy. Ultrasound waves affect tissues via thermal and non-thermal mechanisms. The functional changes in tissues are decided by the frequency and intensity of the ultrasound waves (dosage) and the types of tissues that are exposed to ultrasound. Ultrasound stimulation of the neural system as acoustic neuromodulation has received great interest due to its non-invasive advantage (Kim et al., 2014).

Ultrasound neuromodulation can stimulate or inhibit neural structures, which can be classified as central nervous system or peripheral nervous system influences. Transcranial ultrasound stimulation can target special brain regions and modulate specific neuronal pathways or nuclei. It has been widely researched in basic neuroscience and has been recommended as a potential therapy for neurological diseases (Bystritsky et al., 2011). In terms of the focus on the peripheral nervous system, Wasilczuk et al. (2019) observed that low-intensity focused ultrasound stimulation of the vagus nerve exerted anti-inflammatory effects and significantly reduced tumor factor necrosis-α levels. Recently, ultrasound-induced neuromodulation has gained interest due to its potential to non-invasively modulate ANS activity for the treatment of cardiovascular diseases. Wang et al. (2019a) showed that low-intensity ultrasound stimulation reduced ventricular arrhythmias by modulating sympathetic neural activity in a myocardial infarction canine model.

### Optogenetics in Autonomic Neuromodulation for Cardiovascular Diseases

In 2006, Deisseroth et al. first referred to the word “optogenetics” (Deisseroth et al., 2006). Optogenetics offers a technique to control and monitor the activity of excitable cells by light. This method genetically affects the expression of light-sensitive ion channels, known as opsins, to achieve precise control of targeted cell activity (Deisseroth, 2011). Recently, optogenetics has been widely developed and used in the field of neuroscience and cardiac tissues to control the activity of specific neuron and myocardial populations. It has been applied for the treatment of neurological disorders in experiments (Boyden, 2015). Optogenetics also contributes to further explaining the mechanisms of the initiation, perpetuation, and termination of arrhythmias in cardiac areas (Nussinovitch and Gepstein, 2015; Vogt et al., 2015; Hulsmans et al., 2017).

More recently, optogenetics has enabled the spatially and temporally specific stimulation of cardiac autonomic neurons using light. Photostimulation of neurons expressing the light-gated cation channel channelrhodopsin modulates cardiac autonomic nerve activity and then evaluates the potential cardiovascular changes (Wengrowski et al., 2015; Yamamoto et al., 2015; Gepstein and Gruber, 2017).

Optogenetics selectively stimulates the cardiac ANS by the release of acetylcholine or norepinephrine (Abbott et al., 2013). Yu et al. (2017d) first applied optogenetics to regulate the activity of the left stellate ganglion to prevent ventricular arrhythmias induced by acute myocardial infarction in canine models. Virus was transfected into left stellate ganglion neurons to induce the expression of ArchT proteins. Proper illumination (565 nm) activated ArchT and caused hyperpolarizing currents in the neurons. Optogenetics reversibly suppressed the cardiac sympathetic tone and then exerted a protective role against ventricular arrhythmias associated with autonomic nerve dysfunction.

### Light-Emitting Diode Therapy in the Modulation of the CANS for Cardiovascular Diseases

In the late 1960s, light-emitting diode (LED) therapy was first discovered (Li et al., 2013). LED therapy has been recently introduced into medicine and widely applied in dermatology. LED photomodulation is a non-thermal technology used to modulate cellular activity with light, and photons are absorbed by mitochondrial chromophores in cells. It has been reported that low-intensity LED phototherapy has various protective effects, including inhibiting the inflammatory response and increasing collagen synthesis. Because of the non-invasive therapy with side effects reported in the published literature, LED therapy has gradually been applied in cardiovascular diseases (Gold, 2011; Avci et al., 2014; Capalonga et al., 2016; Sorbellini et al., 2018).

Recently, Wang et al. (2019b) reported that LED therapy reduced post-infarction ventricular arrhythmias by modulating the neuroimmune network. In this study, LED therapy significantly suppressed the activity of the left stellate ganglion and reduced the levels of pro-inflammatory cytokines through the inhibition of microglial activation in the hypothalamic paraventricular nucleus. LED therapy might reduce myocardial ischemia/reperfusion-induced ventricular arrhythmias by attenuating microglial and sympathetic over-activation. They also proved that LED therapy has the same indirect effects of sympathetic activity in a rat model of acute myocardial ischemia/reperfusion injury (Wang et al., 2019c). This indicates that LED affects the ANS through the inhibition of inflammation rather than through direct effects. Currently, there is no evidence to prove that LED can directly activate ANS.

### gammaCore (Non-invasive Cervical Vagus Nerve Stimulation) and Cardiovascular Diseases

gammaCore, a non-invasive cervical vagus nerve stimulator, is already an FDA-approved device for the treatment of primary headache disorders. It is used by sending mild electrical stimulation through the skin to activate the vagus nerve from outside the body. It is programmed for stimulation in cycles for 2 min, and one treatment consists of three cycles (Akdemir and Benditt, 2016; Mwamburi et al., 2017).

This kind of non-invasive neuromodulation has been used to treat cluster headaches (Simon and Blake, 2017). Current clinical evidence shows that gammaCore can reduce the frequency and intensity of cluster headache attacks in some patients (Tassorelli et al., 2018). It can also reduce the need for medication. This is likely to lead to significant quality of life benefits for people living with this condition.

gammaCore™ is a simple-to-use, handheld medical device that enables patients to self-administer discrete doses of non-invasive vagus nerve stimulation (nVNS) therapy. Cost analysis suggests that using gammaCore may lead to cost savings (Mwamburi et al., 2018). However, there is no evidence for the treatment of cardiovascular diseases by gammaCore™. The effect of gammaCore™ on the treatment of cardiovascular diseases needs further study in both experimental and clinical areas. It is an emerging and potential type of neuromodulation for the treatment of cardiovascular diseases.

## Potential Opportunities and Challenges

Non-invasive neuromodulation provides opportunities for better understanding ANS circuits and neurophysiological responses (Boes et al., 2018). It also provides a potential therapeutic target for the treatment of cardiovascular diseases. These kinds of neuromodulation are usually low-cost, portable, and easy to use. Due to the novel and non-invasive approach, it is an attractive therapy for clinical doctors. Patients with non-invasive neuromodulations maybe have a better compliance. Clinical doctor can wirelessly re-set up parameters when patients with chronic pain or movement disorder at home. Non-invasive neuromodulation of the ANS has opened new frontiers for the application of cardiovascular disorders.

However, the road of non-invasive neuromodulations translation in cardiovascular diseases, the same as invasive neuromodulations, is also not flat. There is still a long way to go to translate clinical application to treatment of cardiovascular diseases. For example, divergent results also exist. A dose-response curve is estimated to determine the proper dosage and achieve the most probable benefit in pharmacological trials (Mann and Deswal, 2003). It should also be recommended for invasive and non-invasive neuromodulation (Zannad et al., 2015; Byku and Mann, 2016; DiCarlo et al., 2018). The efficacy of non-invasive neuromodulation might be variable. Multiple reasons might be responsible for the divergent results, including individual differences, protocol parameters (current intensity, frequency, duty cycle), and failure to engage the appropriate neurobiological target. To optimize efficacy and preserve safety, further basic research and clinical studies need to be performed to assess the long-term effects of non-invasive neuromodulation.

## Conclusions

Autonomic dysfunction plays an important role in the process of cardiovascular disorders. Neuromodulation has been proven to be an emerging non-pharmacological approach for the treatment of cardiovascular diseases in basic research and clinical studies. Based on anatomy and nerve denervation, neurostimulation approaches are divided into invasive and non-invasive approaches. Invasive neuromodulation approaches are usually hampered by the potential risks of complications, side effects, increases in electrical impedance, and even perpetual nerve damage. Recently, non-invasive neuromodulation approaches have received great interest in the treatment of cardiovascular diseases. However, due to the limited evidence, further experimental studies and clinical trials are still needed.

## Data Availability Statement

The raw data supporting the conclusions of this article will be made available by the authors, without undue reservation.

## Author Contributions

MC, XL, and SW participated in the study design and drafted the manuscript. SZ and MC were responsible for writing the manuscript. LY, QL, and JT participated in the overall editing and approval of the paper. All authors contributed to the article and approved the submitted version.

## Conflict of Interest

The authors declare that the research was conducted in the absence of any commercial or financial relationships that could be construed as a potential conflict of interest.
